# Integer programming for selecting set of informative markers in paternity inference

**DOI:** 10.1186/s12859-022-04801-z

**Published:** 2022-07-08

**Authors:** Soichiro Nishiyama, Kengo Sato, Ryutaro Tao

**Affiliations:** 1 Graduate School of Agriculture, Kyoto University, Kyoto, Japan; 2 School of System Design and Technology, Tokyo Denki University, Tokyo, Japan

**Keywords:** Optimization, Parentage, Population genetics

## Abstract

**Background:**

Parentage information is fundamental to various life sciences. Recent advances in sequencing technologies have made it possible to accurately infer parentage even in non-model species. The optimization of sets of genome-wide markers is valuable for cost-effective applications but requires extremely large amounts of computation, which presses for the development of new efficient algorithms.

**Results:**

Here, for a closed half-sib population, we generalized the process of marker loci selection as a binary integer programming problem. The proposed systematic formulation considered marker localization and the family structure of the potential parental population, resulting in an accurate assignment with a small set of markers. We also proposed an efficient heuristic approach, which effectively improved the number of markers, localization, and tolerance to missing data of the set. Applying this method to the actual genotypes of apple (*Malus* × *domestica*) germplasm, we identified a set of 34 SNP markers that distinguished 300 potential parents crossed to a particular cultivar with a greater than 99% accuracy.

**Conclusions:**

We present a novel approach for selecting informative markers based on binary integer programming. Since the data generated by high-throughput sequencing technology far exceeds the requirement for parentage assignment, a combination of the systematic marker selection with targeted SNP genotyping, such as KASP, allows flexibly enlarging the analysis up to a scale that has been unrealistic in various species. The method developed in this study can be directly applied to unsolved large-scale problems in breeding, reproduction, and ecological research, and is expected to lead to novel knowledge in various biological fields. The implementation is available at https://github.com/SoNishiyama/IP-SIMPAT.

**Supplementary Information:**

The online version contains supplementary material available at 10.1186/s12859-022-04801-z.

## Background

Parentage assignment is an important issue in various life sciences and has many practical applications. The development of genotyping technology has allowed the wide application of parentage analysis in various research fields, such as ecology, breeding, and reproductive biology. Various statistical methods based on molecular marker genotypes have been developed to analyze parentage [1]. In the past, simple sequence repeat (SSR) markers were used for parentage analysis, but in recent years, SNP markers have become a good choice because they are more reproducible, high-throughput, and have lower evolutionary rates [2–5].

Whatever the method, genotyping typically costs per locus. Hence, a smaller set of markers usually costs less and allows more individuals to be surveyed with the same expense. Since the data generated by high-throughput sequencing technology far exceeds the requirement for parentage analysis, selecting a marker set from thousands or millions of markers across the genome has been attempted for parentage assignment and implemented for many species [4, 6–9], and further reduction in the number of markers will reduce the genotyping cost and would be benefit for a variety of research and application. A common parameter for evaluating the efficiency of a marker set or a marker in parentage inference is the exclusion probability. This is the probability of a randomly chosen pair of individuals being correctly genetically excluded as parents of a randomly chosen individual [10–12]. The exclusion probability depends on the number of alleles for the marker and the allele frequency in the population [10, 13]. However, most SNP markers are biallelic, in which case the exclusion probability depends only on the allele frequency. Marker selection based on the exclusion probability has been used in particular in livestock breeding [6, 7]. In practical studies utilizing SNPs, selection based on minor allele frequency (maf) has also been applied [4, 8, 9].

The exclusion approach is common in parentage analysis; Parentage can be assigned via the exclusion process and by identifying a correct pair of individuals that are not excluded via discrimination tests. However, close familial relationships, such as sibling or parent–offspring relationships, between candidate parents can interfere with the general parentage estimation process [14]. Half of the siblings possess an allele identical by descent (IBD) from a parent, and parent–offspring share at least one IBD allele for every locus, making it difficult to discriminate by exclusion with small number of markers. In practical applications, it is very common that objective populations have such close family structures. A remarkable case is that of fruit tree crops. Because of the perennial life cycle and clonal propagation of fruit tree species, it is common that individuals with parent–offspring relationships are potential parents of seedlings. For example, a small number of elite apple (*Malus* × *domestica*) cultivars have been intensively and recurrently used for the establishment of current germplasm collections [15–17]. Similar breeding schemes have been used for many fruit trees, including Japanese pear [18] and peach [19]. A maf-based selection does not account for the family structure; therefore, to further optimize the marker set for complex populations, such as elite germplasms, it is beneficial for the marker selection to take into account the population’s family structure.

The objective of this study was to develop a computational framework for selecting a small number of markers, from a larger marker pool, that would allow the identification of the paternal parent of the offspring originating from a particular maternal individual (i.e., half-sib family). With the development of targeted SNP genotyping technologies such as KASP, we believed that the combination of novel systematic marker selection could pave the way for the development of cost-effective applications that would allow the analysis in extremely large-scale populations. Here, we targeted the half-sib family because, in many biological problems in which parentage inference is applied, one parent (often the maternal parent) is known before genotyping for the inference, e.g., because the offspring were sampled from the maternal parent. In this study, we used the following three assumptions for the marker selection: (1) the maternal individual is known; (2) the potential paternal individuals have been identified (i.e., a closed population); and (3) the potential paternal individuals have been genotyped. Under these conditions, the problem of minimizing the number of markers to identify the paternal parent of an offspring can be formulated as a binary integer programming problem. In this study, we first obtained an optimized marker set using a basic solver for integer programming problems. We also proposed an efficient heuristic approach that combined the greedy algorithm with a neighborhood search. We then verified that the optimized marker loci worked effectively using real SNP genotype data from an apple germplasm and F_1_ population [15]. Here, apple was chosen as a species for the test study because the familial relationships were well characterized genetically and because of the availability of public data [15].

## Methods

### Formulation of the marker selection problem

**Fig. 1:**
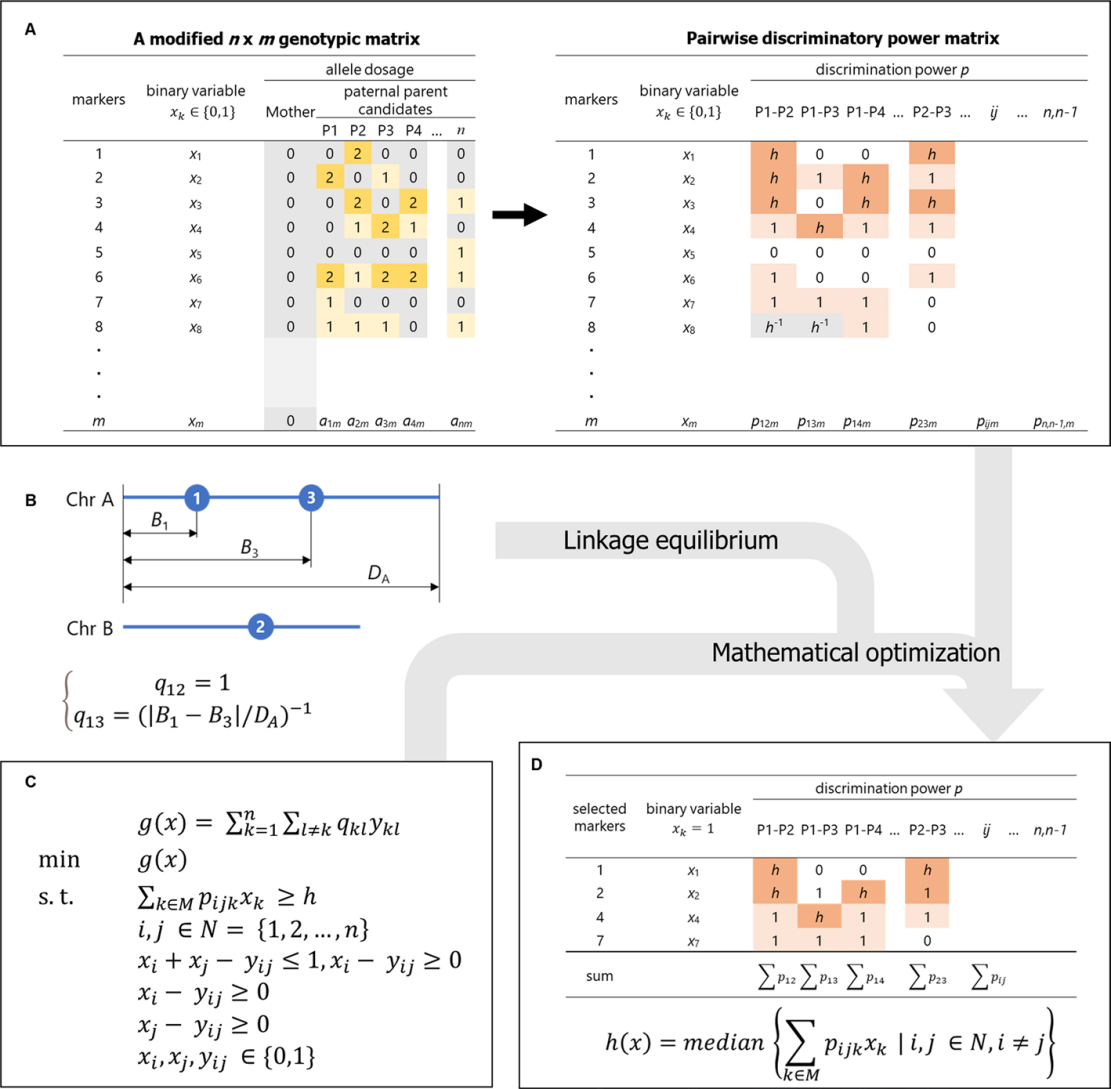
A graphical example of data representation from this study. **A** Genotypic data transformation in this study. First, the users prepare a modified genotype matrix of *n* potential paternal parents for *m* markers in the optimization for a specific maternal parent. Integer values in the left table represent the allele dosages of the markers (${a}_{ik}$). The genotypic data is then transformed to represent the discriminatory power *p* of the markers for each pair of the potential paternal parents, as on the right-side. **B** Representation of the adjacency weight ${q}_{kl}$. Blue circles indicate the physical position of markers. **C** Integer programming formulation in this study. **D** The resultant marker set. Representation of another objective value, “depth” of a marker set *x*, was shown. The minimum requirement of the sum of the discriminatory power *p* for each *ij* pair is defined as *h*, and the “depth”, computed by the function (13), reflects the extent of redundancy in distinguishing each individual in all the pairs of candidate paternal individuals

The problem of selecting a set of markers *x* = (*x*_1_, …, *x*_*m*_) from *m* markers can be formulated as the following binary (0 and 1) integer programming problem (Fig. 1A):

1 $$f\left( x \right) = \mathop \sum \limits_{k \in M} x_{k}$$

2 $${\text{min}}\;\;\;f\left( x \right)$$

3 $$\begin{aligned} & {\text{s}}. {\text{t}}.\;\;\;\;x_{k} \in \left\{ {0, 1} \right\} \\ & \;\;\;\;\;\;\;\;k \in M = \left\{ {1, 2, \ldots ,m} \right\} \\ \end{aligned}$$

where $x_{k}$ represents a binary variable that indicates whether marker *k* is selected. Here the problem is to minimize the sum of $x_{k}$(i.e., the number of selected markers) under constraints (3) and below.

Here, we assumed that the maternal parent was homozygous for all markers with diverse genotypic patterns in the candidate paternal parents (Fig. 1A). The following constraint equation is given for the optimization (2) of discriminating the origin of gametes of *n* candidate paternal individuals:

4 $$\begin{aligned} & {\text{min}}\;\;\;f\left( x \right) \\ & {\text{s}}.{\text{ t}}.\;\;\;\;\mathop \sum \limits_{k \in M} p_{ijk} x_{k} \ge h \\ & \;\;\;\;\;\;\;\;i,j \in N = \left\{ {1, 2, \ldots ,n} \right\} \\ & \;\;\;\;\;\;\;\;i \ne j \\ \end{aligned}$$

where *h* denotes heterozygosity weight, as explained below. Here optimization (2) takes place under the constraint (4) that the sum of the discriminatory power $p_{ijk}$ exceeds the heterozygosity weight *h*.

In the following, we define $p_{ijk}$ as the discriminatory power between individuals *i* and *j* based on the genotype of marker *k*. Because the parental genotype is observed as its gametic genotype in the progeny generation, a pair of individuals with *AA* and *BB* genotypes (*A* and *B* denote alleles in a locus) will always produce gametes with genotypes at the locus that differ between the individuals, thereby yielding the ultimate exclusion inference only with the locus. Therefore, if the markers are arranged so that all pairs of potential paternal parents have the set of the *AA* and *BB* genotypes at least at a particular marker, such a marker set is capable of parentage assignment for every offspring under the aforementioned assumptions. However, when there is a parent–offspring relationship between potential paternal individuals, there will be no *AA* and *BB* genotype pairs between the potential paternal individuals at any loci. Consequently, such individual pairs have a smaller chance of discrimination by exclusion. In the following Eq. (5), we provide different weights to the discriminatory power of each candidate parent-pair using the heterozygosity weight *h* (Fig. 1B):

5 $$\begin{aligned} & p_{ijk} = \left\{ {\begin{array}{*{20}c} {1 \left( {\left| {a_{ik} - a_{jk} } \right| = 1} \right)} \\ {h \left( {\left| {a_{ik} - a_{jk} } \right| = 2} \right)} \\ { h^{ - 1} \left( {a_{ik} = a_{jk} = 1} \right)} \\ {0 \left( {otherwise, \;including\; a_{ik} \;or \;a_{jk} \;is \;missing} \right)} \\ \end{array} } \right. \\ & {\text{where}}\;\;\;\;a_{ik} ,a_{jk} \in \left\{ {0, 1, 2} \right\} \\ & \;\;\;\;\;\;\;\;\;\;\;\;\;\;i,j \in N \\ & \;\;\;\;\;\;\;\;\;\;\;\;\;\;k \in M \\ \end{aligned}$$

where $a_{ik}$ indicates the allele dosage of marker *k* in individual *i*. The constraint (4) and Eq. (5) formalize, even if a paternal candidate pair did not have a homozygous AA or BB pair across the genome, the two parental candidates could be distinguished if there were *h* counts of homozygous (AA or BB) and heterozygous AB pairs.

### The adjacency weight matrix *Q*

Most parentage inference software assumes linkage equilibrium among markers. Extent of linkage disequilibrium is difficult to model because it is sensitive to various parameters, such as population history and recombination frequency [20], but in general, as the physical distance increases, recombination between markers occurs, approaching linkage equilibrium. In addition, marker pairs that locate on different chromosomes are in linkage equilibrium. Therefore, to preferentially select pairs of markers on different chromosomes or pairs that are physically apart, we introduced the following triangular matrix $Q = \left[ {q_{kl} } \right]$ that represents weight based on the physical distance between markers:

6 $$\begin{aligned} & q_{{kl}} = \left\{ {\begin{array}{*{20}l} 1 \hfill & {\left( {c_{k} \ne c_{l} } \right)} \hfill \\ {\left( {\frac{{\left| {B_{k} - B_{l} } \right|}}{{D_{k} }}} \right)^{{ - 1}} } \hfill & {\left( {c_{k} = c_{l} } \right)} \hfill \\ 0 \hfill & {\left( {k \le l} \right)} \hfill \\ \end{array} } \right. \\ & k,l \in M \\ \end{aligned}$$

where ${c}_{k}$ represents the chromosome on which marker *k* is located, ${D}_{k}$ represents the physical length of the chromosome ${c}_{k}$, and ${B}_{k}$ represents the physical position of marker *k* in the chromosome ${c}_{k}$ (Fig. 1C). Using the Eq. (6), we modified the optimization function (1) as follows:

7 $$\begin{aligned} g\left( x \right) & = x^{T} Qx \\ & = \left( {x_{1} x_{2} q_{12} + \ldots + x_{m} x_{m - 1} q_{n,n - 1} } \right) \\ \end{aligned}$$

Because the function (7) is a form of quadratic programming, finding a good solution becomes less straightforward. Here, another dummy variable, $y_{kl} = x_{k} x_{l}$, which makes (7) a linear function, was introduced, as follows:

8 $$g\left( x \right) = \mathop \sum \limits_{k = 1}^{n} \mathop \sum \limits_{l \ne k} q_{kl} y_{kl}$$

9 $${\text{min}}\;\;\;g\left( x \right)$$

10 $${\text{s}}.{\text{t}}.\;\;\;\;\;x_{i} + x_{j} - y_{ij} \le 1, x_{i} - y_{ij} \ge 0, x_{j} - y_{ij} \ge 0, x_{i} ,x_{j} ,y_{ij} \in \left\{ {0,1} \right\}{ }$$

The optimization (9) under the constraints (4) and (10) was calculated by the intlinprog function in MATLAB (version R2020b, The Mathworks Inc.; hereafter, referred to as intlinprog_quad). To verify the effect of the adjacency weight *Q*, optimization calculations were also carried out for the optimization function (1) without *Q* under the constraint Eqs. (2) and (3) (hereafter, intlinprog_single).

### Greedy algorithm

For a more efficient search, we proposed a greedy search method (hereafter, referred to as greedy). We first calculated the effect ${e}_{k}(r)$ of additional marker *k* on the increase in discriminability among individuals, based on the following equation:

11 $$e_{k} \left( r \right) = z_{k} \left( r \right)/\mathop \sum \limits_{l \in M} \left\{ {q_{kl} > 1} \right\}$$

12 $${\text{where}}\;\;\;z_{{k^{\prime}}} \left( r \right) = \mathop \sum \limits_{i \in N} \mathop \sum \limits_{j \in N} {\text{min}}\left( {p_{{ijk^{\prime}}} , r_{ij} } \right)$$

$e_{k} \left( r \right)$ is the sum of the discriminatory power $p_{ijk}$ obtained by the addition of marker *k,* divided by the sum of the adjacency weights matrix *Q* for the existing solution. In (12), we intended to avoid adding the discriminatory effects that were already covered by the existing solution. The residual matrix $r_{ij}$ in (12), with an initial value $r_{ij} = h$, represents the remaining genotype effect required to discriminate between individuals *i* and *j*. In one iteration of the greedy method, the marker with the highest $e_{k} \left( r \right)$ among all the markers is chosen, and $r_{ij}$ was updated. This operation was repeated until all the individuals could be discriminated (i.e., *r* = 0), as shown below:
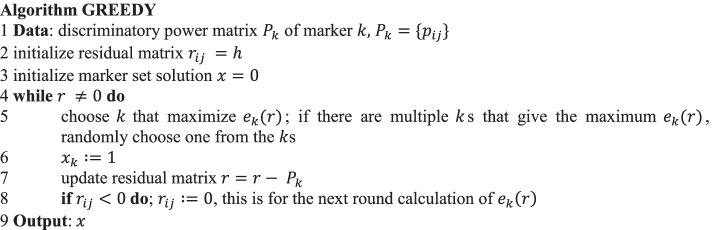


### One/two-flip neighborhood search

In this section, we adopted the neighborhood search algorithm to further improve the solution. In a neighborhood search, the program examines whether a better solution can be obtained on the basis of the evaluation formula for a new solution *x* that is produced by flipping one element of the existing solution. Here, in addition to the optimization function (8), the following evaluation function, calculating “depth” of the discriminatory power of the new solution at the parent-pair basis (Fig. 1D), was employed to avoid early convergence in the neighborhood search:

13 $$h\left( x \right) = median \left\{ {\mathop \sum \limits_{k \in M} p_{ijk} x_{k} | i, j \in N, i \ne j} \right\}$$

The function (13) represents a median of the sum of the discriminatory power between individual *i* and *j* obtained by the solution (marker set) *x*. A marker set with higher value in (13) has a larger buffer of discriminatory power from the minimum requirement and thus is tolerant to missing data.

In a neighborhood search, two types of flips, namely 1- and 2-flips, are performed. In the 2-flip, $x_{s} = 1 \to 0$ at a marker *s* and $x_{t} = 0 \to 1$ at a marker *t* are carried out for all combinations of $s, t \in M$. In the 1-flip, only $x_{s} = 1 \to 0$ was examined for each marker.

A neighborhood search is a time-consuming combinatorial search. To reduce the computational time, we introduced the flip fraction *v* of marker combinations to be flipped in the 2-flip search. Under the formulation, the effect $e_{s} \left( x \right)$ caused by the flip needs to be covered by $e_{t} \left( x \right)$ to satisfy the necessary constraints. In the following algorithm, we first created a correlation matrix for the markers, and for each selected marker (*s*), we chose the $v \in \left[ {0, 1} \right]$ fraction of markers (*t*) in order from the greatest genotypic correlation for the effect evaluation. The neighborhood search was applied to the results of the greedy method and is referred to as greedy + ns.
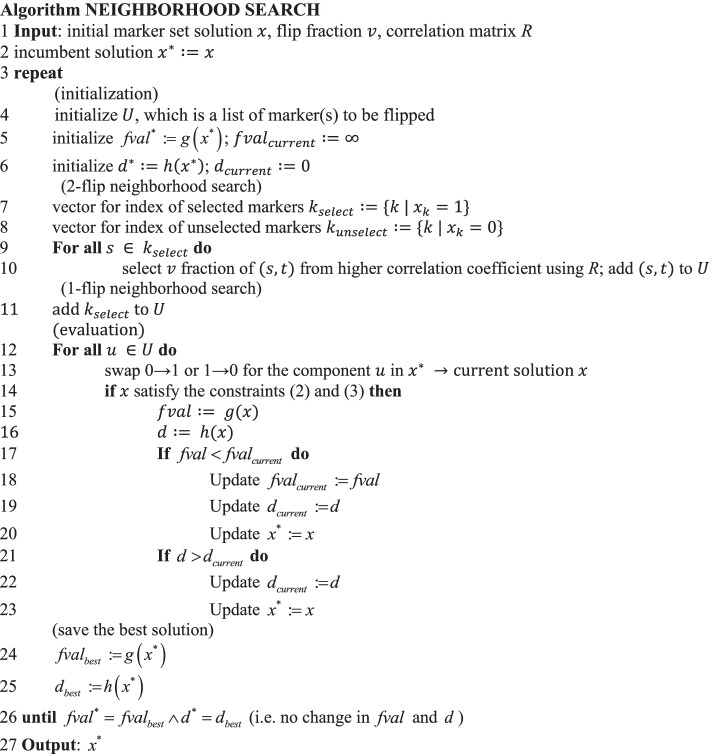


### Apple dataset

For the test study, we used a whole-genome SNP dataset of diploid apple germplasm and F_1_ progeny [15]. The dataset included 1,333 diploid diverse individuals, including various familial relationships between pairs and groups, 46 F_1_ individuals from the cross ‘Fuji’ × ‘Pinova’, and 46 F_1_ individuals from the cross ‘Golden Delicious’ × ‘Renetta Grigia di Torriana’. The germplasm set included four parental individuals of the F_1_ populations.

Here, five types of datasets (Fuji-small, Fuji-middle, Fuji-large, FuPi-family, and GD-large) were created. For the Fuji-small, Fuji-middle, and Fuji-large datasets, ‘Fuji’ was selected as the maternal parent of the half-sib population to be inferred, and the other individuals were considered as paternal parental candidates of the half-sib population. First, to facilitate counting alleles in the paternal parental candidates required in the Eq. (5), all the SNP loci for which ‘Fuji’ was missing or heterozygous were removed, retaining only those that were biallelic in the population and homozygous for ‘Fuji’. For the Fuji-small dataset, we randomly selected 300 SNPs with maf ≥ 0.2 for 30 individuals (29 randomly selected individuals plus ‘Fuji’), and for the Fuji-middle dataset, we selected 301 individuals (300 randomly selected individuals plus ‘Fuji’). The Fuji-large dataset included all 1,333 individuals. For the Fuji-middle and Fuji-large datasets, the initial selection criteria were as follows: marker loci with maf ≥ 0.05, no pair of adjacent 50 loci with R^2^ > 0.5, and no two loci within 10 kb of each other, as determined using PLINK 1.9 [21]. The GD-large dataset was prepared as with the Fuji-large dataset, with the maternal parent set to ‘Golden Delicious’. The GD-large dataset was used to test the multi-family applicability of the proposed optimization method.

In addition, the FuPi-family dataset was created to determine whether the marker selection for paternal parent identification was possible even with a parental population having very similar genotypes. Here, we chose ‘Gala’ as the seed parent and assumed a situation in which 46 ‘Fuji’ × ‘Pinova’ F_1_ individuals plus their parents were randomly mated with ‘Gala’. After selecting biallelic SNP loci homozygous for ‘Gala’, 15,546 loci, meeting the criteria of maf ≥ 0.05 and no two loci were within 10 kb of each other, were selected.

Five datasets were optimized using the proposed methods. Each optimized marker set was used for inferring the paternal parents of the simulated and real offspring genotypes. The genotypes of the simulated offspring were created using an in-house script based on assumptions that all marker pairs of the optimized marker set are in linkage equilibrium and the alleles are transmitted under Mendelian inheritance. We simulated the genotypes of five offspring for each cross between the seed parent (‘Fuji’ or ‘Gala’) and each of the pollen parental candidates. To examine the effect of genotype error on accuracy, we further prepared simulated genotypes that were randomly masked at a given frequency (0.01–20%) and used them for parentage inference.

### Parentage assignment and analysis

Here we tested whether genotypic data of the optimized set of markers, but not of whole markers, makes an inference of true parentage. The maximum likelihood-based software Cervus version 3.0.7 [22] was used to infer paternal parents. Genotypic data of the selected markers for the maternal parent, offspring, and potential paternal parents was formatted for Cervus using a custom script (available in the GitHub repository), and parent of the offspring was inferred by the “Paternity analysis” option in Cervus. When the Cervus’ estimate and the actual paternal parent matched, we labeled the estimation “true positive”, and when they did not match, we labeled the estimation “false positive”. When Cervus did not estimate the paternal parent, the estimation was labeled “unassigned”. To interpret the effects of the optimization, we compared the percentages of correct estimates between the optimized marker set and an equal number of randomly selected markers. The random selection was repeated three times, and the set with the highest proportion of true positives was employed for the comparison with the optimized marker set.

### Computation

The proposed method was implemented in MATLAB, and the calculations were performed using MATLAB 2020b. For evaluations, the programs were run on a CentOS7 machine equipped with two Intel Xeon Gold 5222 (in total 8 cores) running no other job.

## Results

### Effectiveness of integer programming and adjacency weight *Q* for marker selection

First, we tested the validity of the formulation using the Fuji-small dataset (30 individuals and 200 markers). The intlinprog_quad method using the MATLAB intlinprog function produced the optimized solution (Table 1, Additional file 1: Table S1). Except for two markers on chromosome 10, the selected markers were located on different chromosomes, and the two markers on chromosome 10 were more than 20 Mb apart, showing that our formulation provided a marker set that was close to linkage equilibrium.

**Table 1:** Performance of the proposed integer program for optimization of the Fuji-small dataset (30 individuals, 200 markers)

Method	*h*	Time (sec.)	# Markers	$g(x)$	$h(x)$
Intlinprog_quad	8	4.81E+05	13	79.04	22.38
Greedy	8	0.23	17	136.00	27.19
Greedy-neighbor	8	2.01	16	120.00	29.25

Next, we compared the optimization results with and without applying *Q* against the Fuji-middle dataset. We applied the obtained marker set to the real ‘Fuji’ × ‘Pinova’ F_1_ population and tested whether the pollen parent could be inferred to be ‘Pinova’. The *Q*-applied greedy + ns method selected 23 markers and correctly assigned the parentage for all F_1_ individuals with the “most likely” threshold. However, the intlinprog_single method, which did not apply *Q*, failed to correctly assign 7.0% (3/46) of the F_1_ individuals at the same “most likely” threshold (Table 2). In particular, using intlinprog_single, false positives occurred regardless of the threshold values. In summary, we confirmed that the discriminability was increased by the application of *Q*.

**Table 2:** Effect of the distance weight *Q* on the success of parentage inference

Optimization			'Fuji' × 'Pinova' F1 (*N* = 46)								
Method	*h*	# Markers	Strict (95% confidence)			Relaxed (80% confidence)			Most likely		
			TP	FP	Unassigned	TP	FP	Unassigned	TP	FP	Unassigned
intlinprog_single, without applying distance weight *Q*	8	24	37	1	8	43	1	2	43	3	0
greedy + ns, flip-fraction v = 0.2		23	41	0	5	44	0	2	46	0	0

Optimization was performed on the Fuji-middle dataset (300 individuals and 11,954 markers). TP and FP represent true and false positives, respectively

### Effectiveness of the greedy method and neighborhood search

**Fig. 2:**
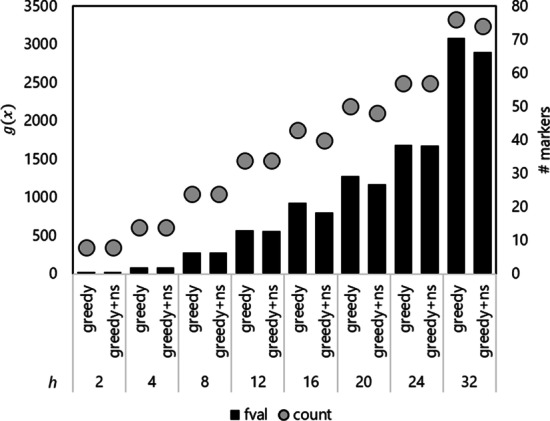
Optimization of the Fuji-middle dataset. Fuji-middle dataset consisted of 301 individuals and 11,954 markers, and the dataset was optimized using a neighborhood search (ns) as well as the greedy method (greedy). The optimized value by the function (8) and the number of markers are shown as black bars and red circles, respectively. Different values for the heterozygosity weight *h* were tested for the optimization

The application of the greedy method significantly reduced the time required to determine a solution, and the addition of a neighborhood search resulted in an improved solution (Table 1). Although the optimization results using the heuristic methods were slightly inferior to the result of the intlinprog_quad method, the proposed heuristics were computationally less expensive and appeared to be effective for large-scale data. In fact, the solution for the Fuji-middle set was not obtained in our machine by using the intlinprog method, whereas the greedy method and the neighborhood search yielded a solution in a reasonable time (Additional file 1: Table S2). When a large number of markers was targeted, the adoption of a neighborhood search improved both the optimization function (8) and the number of markers (Fig. 2). With the adoption of the flip fraction *v*, solutions that were better than searching all the combinations were obtained in less time for our dataset (Additional file 1: Table S2).

### Effectiveness and reasonable choice of the heterozygosity weight *h*

**Fig. 3:**
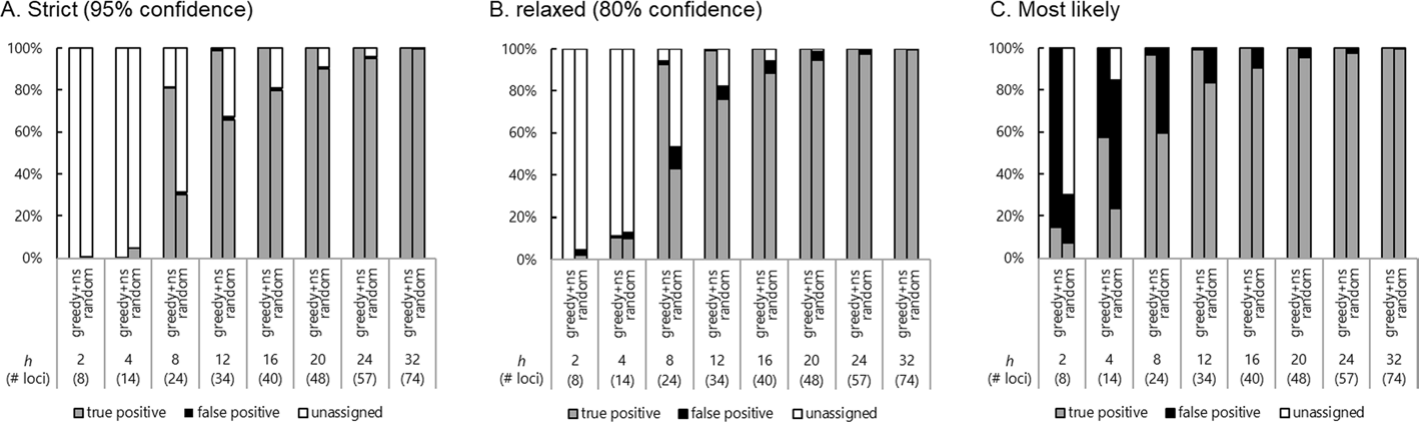
Comparison of the effects of the heterozygosity weight *h* on the paternal parent inference. The marker set optimized for the Fuji-middle dataset (greedy + ns) was compared with a marker set containing an equal number of random markers (random). The random selection was repeated three times, and the set with the highest proportion of true positives was shown. The discriminatory criteria “strict” (**A**), “relaxed” (**B**), and “most likely” (**C**), which are the outputs of the Cervus software, were applied

Increasing the value of *h* increased the value of $g(x)$ and the number of markers selected (Fig. 2). Of the three thresholds used for the Cervus parentage inference, "most likely" produced the largest count of true positive inferences, but also produced large number of false positives (Fig. 3). As *h* was increased and more markers were used, the accuracy of the inference also increased. Applications of various *h* on the Fuji-middle dataset revealed that *h* ≥ 16 produced fully discriminative marker sets at any of the thresholds (Fig. 3), whereas, for the random marker set, false positives were observed even with a random marker set consisting of 74 markers, the same number of markers as for *h* = 32. In particular, *h* = 12 yielded a set of 34 markers which achieved ≥ 99% true positives using all three thresholds applied in the inference for a simulated population.

Next, we optimized a marker set for the simulated population (Gala × FuPi family). The paternal candidates of this population showed extremely high allele sharing, and thus, it should have been difficult to identify the paternal parents. However, using the optimized marker set with *h* ≥ 12, we were able to identify the paternal parents in more than 98% of the combinations (Table 3). The discriminatory rate for the parental cultivars (‘Fuji’ and ‘Pinova’) was particularly low, but a correct identification rate of more than 93% was obtained at *h* = 16. The F_1_ siblings could be accurately discriminated with *h* ≥ 8.

**Table 3:** Success rate of pollen parent identification using a marker set optimized for a simulated population Gala × FuPi family.

optimization				Gala × FuPi family simulated population																	
				total [*N* = 4800 (100 × 48)]						[Gala × Fuji] and [Gala × Pinova] [*N* = 200 (100 × 2)]						[Gala ×FuPi siblings] [*N* = 4600 (100 × 46)]					
*h*	# Markers	*g*(*x*)	*h*(*x*)	Strict (%)		Relaxed (%)		Most likely (%)		Strict (%)		Relaxed (%)		Most likely (%)		Strict (%)		Relaxed (%)		Most likely (%)	
				TP	FP	TP	FP	TP	FP	TP	FP	TP	FP	TP	FP	TP	FP	TP	FP	TP	FP
8	16	120.04	27.13	92.21	0.94	95.71	2.52	96.69	3.10	16.00	16.00	26.00	44.50	43.50	52.00	95.52	0.28	98.74	0.70	99.00	0.98
12	23	253.50	47.42	98.56	0.40	99.23	0.75	99.23	0.75	72.50	8.50	82.50	17.00	82.50	17.00	99.70	0.04	99.96	0.04	99.96	0.04
16	27	355.86	59.56	99.73	0.10	99.83	0.15	99.83	0.15	93.50	2.50	96.00	3.50	96.00	3.50	####	0	100	0	100	0

The FuPi dataset (15,546 markers) consisted of 46 siblings from a cross 'Fuji' × 'Pinova', and their parents 'Fuji' and 'Pinova'. The marker set was optimized for the FuPi dataset. Pollen parent identification was performed using the simulated marker genotype of the optimized marker set. TP represents true positive, and FP represents false positive (wrong assignment). The remainder is the fraction of unassigned individuals

### Optimization of the large datasets and its application to a real F_1_ population

The Fuji-large dataset (1333 individuals and 12,229 markers), including all diploid lines in the population described by Muranty et al*.* [15], was optimized, yielding an estimation accuracy equivalent to that of the Fuji-middle dataset (Additional file 1: Tables S3 and S4). Using the real F_1_ population ‘Fuji’ × ‘Pinova’, we obtained fully correct assignments of the parentage with a threshold of “most likely” for *h* = 8 and all three thresholds for *h* = 12 (Table 4). In addition, no false positives were observed under any of the conditions tested. We also confirmed that masking of genotype data up to 1%, which is above the typical genotype error rate, did not significantly reduce the accuracy of paternity inference (Additional file 1: Table S3).

**Table 4:** Optimization of the Fuji-large dataset and test application to determine the parentage inference of a real F1 population ‘Fuji’ × ‘Pinova’

*h*	optimization			'Fuji' x 'Pinova' F1 (*N* = 46)								
	*g*(*x*)	# Markers	*h*(*x*)	Strict			Relaxed			Most likely		
				TP	FP	Unassigned	TP	FP	Unassigned	TP	FP	Unassigned
8	#####	30	38.88	35	0	11	38	0	8	46	0	0
12	#####	41	69.75	46	0	0	46	0	0	46	0	0

Strict (95% confidence), relaxed (80% confidence), and “most likely” thresholds were applied for the paternity inference

*TP* True positive, *FP* false positive

Selected markers tended to have higher maf than the genome-wide average (Additional file 2: Figure S1). In the optimized sets for the Fuji-large dataset, there are 14 intersects between 30 markers in the *h* = 8 set and 41 markers in the *h* = 12 set; the 14 intersect markers have high maf (0.387–0.493), whereas remaining several have maf lower than the genome-wide average (Additional file 2: Figure S1).

To test the multi-family applicability of the proposed optimization method, the GD-large dataset was optimized for ‘Golden Delicious’ as a maternal parent. The optimization yielded similar values of the optimization variables as in the optimization of Fuji-large dataset (Additional file 1: Tables S5 and S6). The optimized set was applied in the real F_1_ population, ‘Golden Delicious’ × ‘Renetta Grigia di Torriana’, and found to produce fully correct inference of their paternal parent to be ‘Renetta Grigia di Torriana’, with a threshold of “relaxed” for *h* = 8 and all three thresholds for *h* = 12 (Additional file 1: Table S5).

## Discussion

### Integer programming formulation and its applications

In this study, we proposed a novel computational framework to select markers for parentage assignment on the basis of a binary integer programming formulation. As shown in Fig. 3, the proposed method yielded marker sets that produced fewer false positives and required fewer markers than standard methods. The adjacency weight *Q* allowed the preferential selection of markers approaching linkage equilibrium, whereas applying the heterozygosity weight *h* enabled the estimation of a population with a close family structure. The beneficial effects of these parameters on the estimation accuracy were demonstrated using simulated and actual progeny populations in this study (Tables 2 and 3).

The present optimization method was designed as being identity by state (IBS)-driven; the solution heavily relies on IBS in the paternal candidate population. This point allowed for simple implementation and made it possible to apply the method without taking into account the complex ancestry (descent) relationships prevalent in wild and breeding populations. As proof, optimization effectively worked for a paternal parent population that included only individuals with first- or second-degree relationships (the FuPi family), whereas the number of markers required was higher due to its high allele sharing (Table 3).

The method developed in this study has various applications. Breeding is one practical field in which parentage information is fundamental for its productivity. For example, in aquaculture fish breeding, marker-based parentage analyses are essential for breeding program success [12, 23]. Additionally, molecular ecological studies extensively analyzed parentage to infer reproductive characteristics, and marker-based analyses are actively being conducted [14, 24]. The three assumptions in the present formulation (see “Introduction”) may now be satisfied in many biological studies, and in those cases, the proposed method can be used directly.

The method proposed in this study will enable low-cost parentage identification, which is advantageous in large-scale analyses. For instance in fruit tree species, inexpensive pollen parent identification applications may maximize the efficiency of programed crossings in a single year and reduce breeders’ labor during the flowering season, especially for species, such as litchi and mango, that have very low fruit set rates and are extremely difficult to cross artificially. Additionally, it may be used in many genetic analyses, including characterization of genetic factors that strongly control the success of crossings in certain combinations, such as self-incompatibility [25, 26]. In addition, identifying the pollen parents of many seeds (fruits) would increase our understanding of pollination-related flower–insect interactions at the field level, which would ultimately lead to optimized pollination strategies and benefit crop production.

In the presented implementation of the *Q* matrix, the physical distance between markers was employed to the weights when the marker pair was located on the same chromosome; however, this should inherently depend on the genetic distance. Although there is no guarantee that the genetic maps of all individuals in a population are identical, if a genetic map of some individuals is available, as in the case of apple [27], this may be used as a representative of the weights. This study showed that linear weight for physical distance could be a good alternative, and the efficient optimization was validated using the real hybrid offspring (Tables 2 and 4, Additional file 1: Table S5).

### Selection of hyperparameters

In this study, we investigated various values of the heterozygosity weight *h,* which was introduced to analyze populations with complex familial relationships. In the Fuji-middle and the Fuji-large datasets, practical sets of markers were obtained for *h* ≥ 8 (Fig. 3). A significant proportion of the markers selected with different *h* values were shared, and these shared markers exhibited high maf (Additional file 2: Figure S1). This implies that markers with higher information content are more preferentially employed, whereas markers with less information content are required to be included to satisfy the remaining constraints. The optimal *h* enabling an accurate parentage inference appeared to be greater when the paternal candidate population had a closer familial relationship (Table 3). The optimal *h* also depended on the desired performance. The best practice at the moment is to conduct simulations similar to the one performed in this study for each population and set *h* in accordance with the user’s preferences.

In the neighborhood search, we introduced the flip fraction *v* to reduce the computational time. In our 2-flip neighborhood search, the flipping pair (*s*, *t*) must satisfy the given constraints when *s* is replaced by *t*. In fact, the number of pairs satisfying the constraints was very limited. In this study, searching approximately 5%–20% of the markers that correlated with the genotype of *s* yielded the best solution and could yield a solution even better than searching the whole data (Additional file 1: Table S2), depending on the conditions, such as family structure and the initial marker number.

## Conclusions

Here, we present a new tool for selecting informative markers for paternity inference, using a binary integer programming formulation. Since the data generated by high-throughput sequencing technology far exceeds the requirement for parentage assignment, we thought that a cost-effective application could be generated by combining the systematic marker selection with targeted SNP genotyping such as KASP. We propose two key hyperparameters that address the typical problems arising in marker set for parentage inference. In addition to the proposed solver-based approach, we developed a greedy iterative heuristic and neighborhood search implementations, allowing the efficient calculation of the proposed problem. The test results using simulated and real hybrid populations of apple genotypes validated the effectiveness and computational-efficiency of our systematic approach.

## Supplementary Information

**Additional file 1.** Supplementary tables.

**Additional file 2.** Supplementary figure.

## Data Availability

The implementation and all analytical codes used in this study are available at https://github.com/SoNishiyama/IP-SIMPAT.

## Abbreviations

SSR: Simple sequence repeat
SNP: Single nucleotide polymorphism
IBD: Identical by descent
IBS: Identity by state
