# Supplementary material for: Integer programming for selecting set of informative markers in paternity inference
Source: BMC Bioinformatics. 2022 Jul 8;23:265. doi: 10.1186/s12859-022-04801-z (PMC9264695; doi:10.1186/s12859-022-04801-z)
Supplement: Supplementary file 2 — Additional file 2. Supplementary figure. [file 12859_2022_4801_MOESM2_ESM.docx]

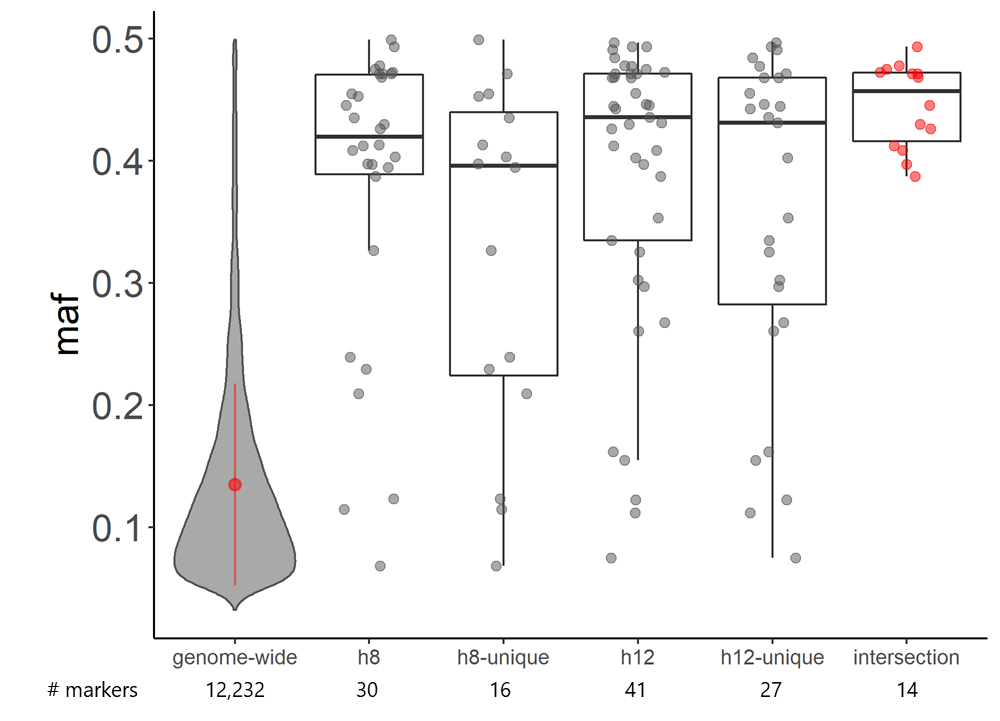


Figure S1. Comparison of minor allele frequency of the optimized marker sets. “h8” and “h12” represent the optimized set for the Fuji-large dataset, with *h* = 8 and *h* = 12, respectively (Table 4, Additional file 1: Table S4). “intersection” represents the commonly selected markers in the "h8" and "h12" sets, while "h8-unique" and "h12-unique" represent the rest. “genome-wide” represents all the markers in the Fuji-large dataset. Red dot and bars in the “genome-wide” plot represent mean and ∓SD, respectively.
